# Primary Neuroendocrine Carcinoma of the Gallbladder - A Case Report

**DOI:** 10.34172/aim.2023.62

**Published:** 2023-07-01

**Authors:** Ankita Majumder, Maruti Arjun Dhakane

**Affiliations:** ^1^Department of Pathology, Apollo Hospitals, Bilaspur, Chattishgarh, India

**Keywords:** Gallbladder, Neuroendocrine tumor, Small cell neuroendocrine carcinoma

## Abstract

Gallbladder neuroendocrine tumors remain relatively rare in the clinical setting. They are generally asymptomatic and reported as incidental findings. The diagnosis is exclusively made by histopathological and immunohistological examination according to the recent WHO guidelines. Here, we present the case of a 58-year-old woman with small-cell gallbladder neuroendocrine carcinoma.

## Introduction

 Neuroendocrine tumors are an epithelial neoplasm with morphological and immunohistochemical neuroendocrine differentiation. They can arise in almost all organs; however, primary small cell neuroendocrine carcinoma of the gallbladder is a very unusual and aggressive malignant tumor, with ~40 cases reported in the medical literature.^1^ They are generally seen in elderly females with cholelithiasis, with cigarette smoking as a significant risk factor.^2^

## Case Report

**Figure 1:**
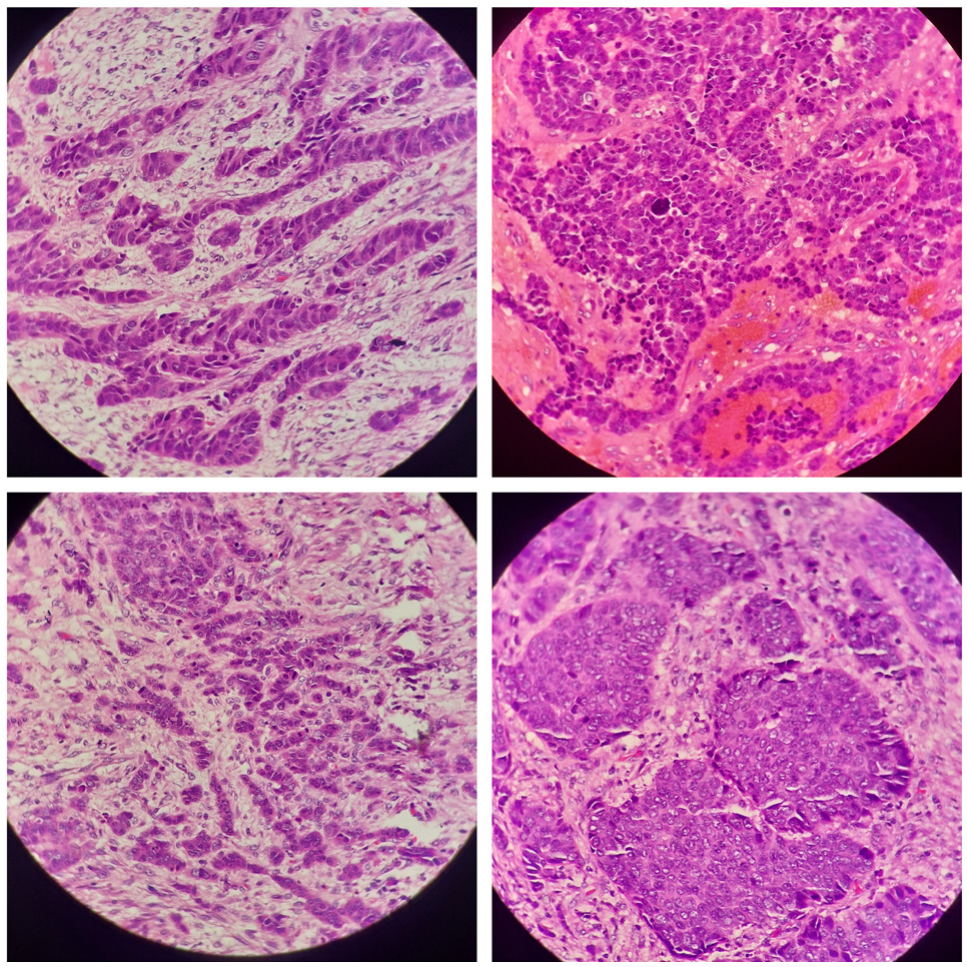
Neuroendocrine carcinoma Consisting of sheets and nests of poorly differentiated, small- to medium-sized, monomorphic, atypical cells and having enlarged hyperchromatic nuclei, speckled chromatin, inconspicuous nucleoli

**Figure 2:**
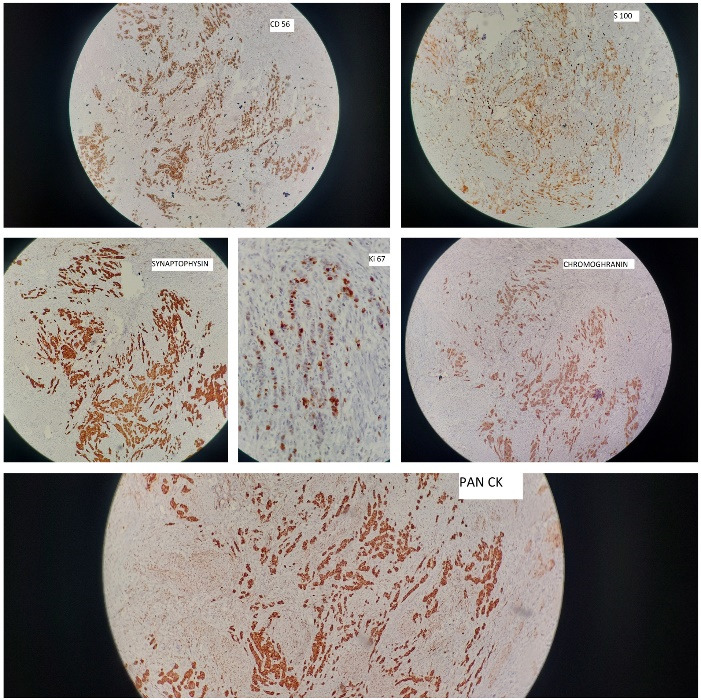
IHC panel shows immune-reactivity for pancytokeratin, CD56, S100, chromogranin and synaptophysin. Ki67 revealed high proliferative index

 A 58-year-old woman from central India presented with chronic, intermittent, right upper-quadrant pain, accompanied by nausea and vomiting. Based on clinical and radiological evaluations, cholelithiasis was suspected and laparoscopic cholecystectomy was performed for her. The specimen was received for routine histopathological examination; on gross examination, a polypoidal and infiltrative gray white necrotic mass lesion was identified located at the neck of the gallbladder, measuring 2.5 × 1.5 × 1.3 cm along with multiple blackish green pigmented gall stones. Routine histology revealed the gallbladder wall to be infiltrated by malignant tumor extending to the muscular layer and perimuscular connective tissue and consisting of sheets and nests of poorly differentiated, small- to medium-sized, monomorphic, atypical cells and having enlarged hyperchromatic nuclei, speckled chromatin, inconspicuous nucleoli, increased mitoses (6‒8/hpf) and scanty cytoplasm admixed with extensive coagulative tumor necrosis (Figure 1). Perineurial invasion, as an adverse indicator, was also identified. The immunohistochemical profile was relatively uniform, with immune-reactivity for pancytokeratin, CD56, S100, chromogranin and synaptophysin. Ki67 revealed high proliferative index (Figure 2). These classical morphological features, along with the uniform staining pattern, confirmed the neuroendocrine lineage of the lesional cells and the final diagnosis was confirmed as primary small cell neuroendocrine carcinoma of the gallbladder.

## Discussion

 Neuroendocrine carcinomas within the gallbladder are quite rare, accounting for only 2% of all gallbladder primary malignancies. They have been reported predominantly in females in the age group 38–81 years.^3,4^ The frequency of carcinoid syndrome is only < 1%.^5^ Normally, no neuroendocrine cells exist in the gallbladder and the origin of these tumors is controversial. Most of these tumors are diagnosed incidentally after cholecystectomy for cholelithiasis. Many researchers have proposed that chronic inflammation due to cholesterol gallstones leads to metaplastic change of the gallbladder epithelium to neuroendocrine cells and increases the risk of neuroendocrine carcinoma in the gallbladder by manifolds. Sakamoto et al found that epithelial metaplasia occurred in 11.7% of patients with cholelithiasis and expressed positive staining for neuroendocrine antigens like chromogranin A in 83.3% and serotonin in 50.0% of metaplastic cells.^6^ Other researchers insist on transformation from gallbladder adenocarcinoma. Mutual conversion of neuroendocrine and adenocarcinoma has been documented.^6^ Neuroendocrine carcinomas usually progress rapidly along with early liver invasion and lymphatic metastases. Treatment varies from simple cholecystectomy to extensive radical resection. Radiotherapy and chemotherapy could help improve the patient’s survival. To conclude, the primary neuroendocrine carcinomas of the gallbladder have a low incidence rate, no specific clinical presentation, and poor prognosis. Histopathological and immunohistochemical examination is necessary for diagnosis.

## Conclusion

 Neuroendocrine carcinoma gall bladder is a very rare and aggresive neoplasm with poor prognosis. So, its important to promptly diagnose this type of malignancies. Immunohistochemistry can be a particularly useful tool in this process to ensure timely diagnosis and start of treatment.
